# Supplementary material for: Genetic Associations of Novel Behaviour Traits Derived from Social Network Analysis with Growth, Feed Efficiency, and Carcass Characteristics in Pigs
Source: Genes (Basel). 2022 Sep 8;13(9):1616. doi: 10.3390/genes13091616 (PMC9498370; doi:10.3390/genes13091616)
Supplement: Supplementary file 1 [file genes-13-01616-s001.zip › genes-1887073-supplementary.pdf]

Table S1. Descriptive statistics of the social network analysis traits.

Traits	Average	SD	Min	Max
Degree centrality	2	2.14	0	17
Weighted degree centrality	128	355	0	3506
Closeness centrality	0.0011	0.0011	0.0000	0.0045
Eigenvector centrality	0.1649	0.3069	0.0000	1.0000
Betweenness centrality	8	14	0	86
Clustering coefficient	0.0851	0.2035	0.0000	1.0000

Table S2. Spearman rank correlations (confidence interval) among the phenotypic values of the social network traits of aggressive behaviour.

Trait	Weighted degree	Betweenness centrality	Closeness centrality	Eigenvector centrality	Clustering coefficient
Degree	0.90(0.88, 0.91)	0.76(0.73, 0.79)	0.67(0.63, 0.71)	0.74(0.69, 0.77)	0.60(0.55, 0.65)
Weighted degree		0.59(0.53, 0.64)	0.54(0.48, 0.59)	0.79(0.76, 0.82)	0.50(0.44, 0.55)
Betweenness centrality			0.41(0.35, 0.47)	0.48(0.42, 0.53)	0.38(0.32, 0.44)
Closeness centrality				0.54(0.48, 0.59)	0.36(0.29, 0.42)
Eigenvector centrality					0.39(0.33, 0.45)

Table S3. Spearman rank correlations (confidence interval) between the phenotypic values of social network analysis traits and performance traits.

Trait	Degree	Weighted Degree	Betweenness centrality	Closeness Centrality	Eigenvector Centrality	Clustering Coefficient
TDG	0.19(0.18, 0.25)	0.17(0.10, 0.24)	0.13(0.06, 0.20)	0.15(0.08, 0.22)	0.14(0.07, 0.21)	0.15(0.08, 0.21)
LDG	0.16(0.09, 0.20)	0.15(0.08, 0.22)	0.10(0.03, 0.17)	0.15(0.08, 0.22)	0.12(0.05, 0.18)	0.12(0.05, 0.19)
DFI	0.05(-0.02, 0.12)	0.05(-0.02, 0.12)	0.03(-0.04, 0.10)	0.07(-0.002, 0.13)	0.03(-0.04, 0.10)	0.06(-0.01, 0.12)
FE	-0.18(-0.25, -0.11)	-0.16(-0.23, -0.09)	-0.13(-0.19, -0.06)	-0.12(-0.18, -0.05)	-0.15(-0.22, -0.09)	-0.14(-0.20, -0.06)
FBW	0.16(0.09, 0.22)	0.16(0.09, 0.22)	0.10(0.03, 0.17)	0.15(0.08, 0.22)	0.12(0.05, 0.19)	0.12(0.05, 0.19)
HCW	0.13(0.06, 0.2)	0.12(0.05, 0.19)	0.07(0.002, 0.14)	0.15(0.08, 0.22)	0.09(0.01, 0.16)	0.10(0.03, 0.169)
BF	0.01(-0.06, 0.08)	0.01(-0.06, 0.08)	-0.05(-0.11, 0.02)	0.13(0.06, 0.19)	-0.01(-0.08, 0.06)	0.08(0.01, 0.149)
Loin	0.03(-0.04, 0.1)	0.04(-0.03, 0.11)	0.03(-0.04, 0.09)	0.04(-0.03, 0.11)	0.06(-0.007, 0.13)	-0.02(-0.09, 0.05)

TDG = Test daily gain (g/d), **LDG** = lifetime daily gain (g/d), **DFI** = daily feed intake (g/d), **FE** = feed efficiency, **FBW**= final body weight (kg), **HCW** = hot carcass weight (kg), **BF** = back fat (mm), **LD** = loin depth (mm).
